# Case Report: Flossing intervention for chronic upper limb spasticity after childhood-onset stroke

**DOI:** 10.3389/fstro.2026.1952839

**Published:** 2026-09-30

**Authors:** Shuichi Sasaki, Naonobu Takahira, Yuzo Ono, Keiichiro Aoki, Sora Kurosaki, Marina Moteki, Risa Fujita, Tomonori Kenmoku

**Affiliations:** 1 Department of Rehabilitation, Kitasato University Hospital, Sagamihara, Japan; 2 School of Allied Health Sciences, Kitasato University, Sagamihara, Japan; 3 Department of Occupational Therapy, School of Rehabilitation, Tokyo Professional University of Health Sciences, Tokyo, Japan; 4 Department of Orthopedic Surgery, Kitasato University, School of Medicine, Sagamihara, Japan

**Keywords:** flossing intervention, rehabilitation, spasticity, stroke, upper limb

## Abstract

**Background:**

Upper limb spastic paralysis after stroke often limits functional use of the affected hand and restricts activities of daily living, particularly in cases with long-standing impairment following childhood-onset stroke. Although various interventions for spasticity have been reported, many require specialized equipment or invasive procedures, which may limit long-term and home-based use. Flossing intervention, which involves compressive wrapping with an elastic band combined with movement, has recently been introduced in musculoskeletal practice; however, its application for post-stroke spasticity remains unclear.

**Case presentation:**

An adolescent girl in her teens (female, Asian) presented with long-standing right upper limb spastic paralysis following childhood-onset cerebral infarction associated with moyamoya disease. At baseline, she demonstrated marked distal spasticity, limited active wrist extension, and restricted functional use of the affected hand. A flossing intervention was applied to the distal forearm and elbow region, consisting of compressive wrapping followed by passive twisting and active movements. The intervention was performed both in outpatient therapy and as a home-based program. After 6 months of continuous intervention, Modified Ashworth Scale scores decreased, upper limb motor function assessed using the Fugl–Meyer Assessment improved, and real-world use of the affected hand assessed using the Motor Activity Log increased. Active wrist extension range of motion improved from −20° to 40°, and functional bilateral hand use, including button fastening, became possible. The patient reported improved ease of hand movement and high satisfaction, and functional gains were maintained during follow-up.

**Conclusions:**

This case suggests that flossing intervention may be a feasible, non-invasive, and home-based treatment option for improving upper limb function in patients with long-standing post-stroke spastic paralysis following childhood-onset stroke. Further studies are required to clarify its efficacy and appropriate indications.

## Introduction

Upper limb spastic paralysis is a common sequela after stroke and frequently interferes with voluntary movement, functional hand use, and activities of daily living (Wissel et al., 2013; Pandyan et al., 2005; Urban et al., 2010) Spasticity of the wrist and fingers markedly limits grasping, releasing, and bimanual tasks, resulting in long-term restrictions in participation and quality of life. These limitations are often more pronounced in patients who experience stroke during childhood, as neurological impairment persists through critical periods of motor development.

Various interventions have been proposed for the management of post-stroke spasticity, including botulinum toxin injections, non-invasive brain stimulation such as repetitive transcranial magnetic stimulation and transcranial direct current stimulation, and extracorporeal shock wave therapy (Esquenazi et al., 2013; Wang et al., 2022; Cabanas-Valdés et al., 2020). Although these approaches have demonstrated clinical effectiveness, their use may be constrained by limited duration of effects, the need for specialized equipment or expertise, and difficulties in maintaining long-term or home-based treatment.

Flossing intervention is a relatively novel technique that involves compressive wrapping of a limb using an elastic band, combined with passive and active movements (Konrad et al., 2021; Vogrin et al., 2021; Driller and Overmayer, 2017). Originally introduced in sports and musculoskeletal rehabilitation, flossing has been reported to improve joint range of motion, reduce pain, and enhance soft tissue mobility. Proposed mechanisms include transient ischemia followed by reactive hyperemia, modulation of sensory receptors, and improved gliding of muscles, tendons, and fascia.

Recent studies have suggested that flossing may influence neuromuscular function through its effects on circulation and sensory input; however, clinical evidence supporting its use in patients with neurological disorders remains limited. In particular, there are few reports describing the application of flossing intervention for upper limb spastic paralysis after childhood-onset stroke.

Therefore, the purpose of this case report is to describe the functional outcomes of a flossing intervention applied to an adolescent girl with long-standing upper limb spastic paralysis following childhood-onset stroke.

## Case history / examination

An adolescent girl in her teens (female, Asian), was referred for occupational therapy to improve upper limb function. She was left-handed and had a history of childhood-onset cerebral infarction associated with moyamoya disease. During early elementary school, she suddenly developed right hemiparesis and subsequently underwent cerebral revascularization surgery in the left temporal region. Postoperatively, she experienced a new extensive infarction involving the left cerebral hemisphere. Following the stroke, she received long-term outpatient management for epilepsy, which was well controlled with medication, as well as speech therapy. Speech therapy had been discontinued several years before the present intervention. No occupational therapy intervention for the upper limb had been provided before the present referral, partly due to psychological aversion related to previous medical experiences. Occupational therapy was initiated at the present referral, and the flossing intervention was introduced at the beginning of the occupational therapy program. At the time of referral, she was able to communicate effectively, demonstrated intact comprehension, and showed no cognitive impairment. Academic performance was at an average to above-average level, and she was preparing for university entrance examinations. Magnetic resonance imaging (fluid-attenuated inversion recovery sequence) revealed chronic infarction extending from the left frontal lobe to part of the temporal lobe (Figure 1). Neurological examination demonstrated spastic paralysis predominantly affecting the distal muscles of the right upper limb.

**Figure 1:**
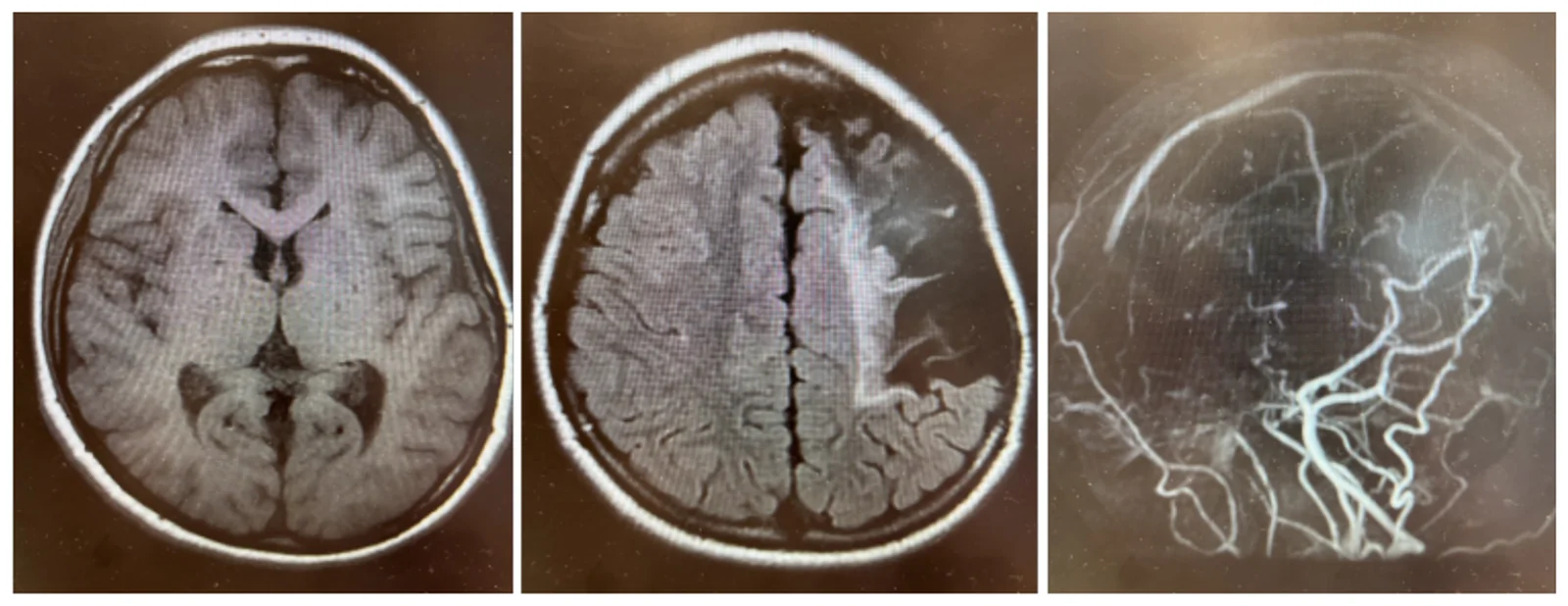
Brain magnetic resonance imaging and magnetic resonance angiography. Fluid-attenuated inversion recovery (FLAIR) images demonstrate chronic infarct lesions involving the left frontal lobe and partially extending into the temporal lobe following childhood-onset stroke. Magnetic resonance angiography (MRA) demonstrates the cerebral vasculature.

Baseline assessment of spasticity using the Modified Ashworth Scale revealed a score of 1+ for the elbow flexors, 2 for the forearm pronators, and 2 for the finger flexors (Bohannon and Smith, 1987). Upper limb motor function assessed using the Fugl-Meyer Assessment was 43 out of 66 points, with marked impairment in distal components (Fugl-Meyer et al., 1975). Active range of motion of wrist extension was limited to −20 °. The thumb showed adduction at the carpometacarpal joint with hyperextension at the metacarpophalangeal joint, consistent with a Z deformity.

In daily activities, the affected hand was used only as an assistive limb. Functional use of the hand was limited, particularly for tasks requiring finger opening and precise manipulation. The Motor Activity Log revealed low scores for both amount of use (1.42) and quality of movement (1.71), indicating restricted real-world use of the paretic upper limb.

Written informed consent for participation and publication, including supplementary video materials, was obtained from the patient and her guardian. The clinical course and timing of the intervention and assessments are summarized in Table 1.

**Table 1:** Timeline of the clinical course, intervention, and assessments.

Time point	Clinical course, intervention, and assessment
Early elementary school	Childhood-onset cerebral infarction associated with moyamoya disease; cerebral revascularization surgery followed by an extensive infarction involving the left cerebral hemisphere
Thereafter	Long-term outpatient management for epilepsy; speech therapy was provided after the stroke but had been discontinued several years before the present intervention; no upper limb occupational therapy
Baseline (start of occupational therapy)	Initial upper limb assessment; occupational therapy initiated and flossing intervention introduced
During the 6-month intervention period	Home-based flossing performed at least twice daily, typically in the morning and evening, with two to three sets per session; outpatient supervision approximately once every 2 months
6 months	Follow-up assessment of upper limb motor function, resistance to passive movement, range of motion, and functional use of the affected upper limb

## Differential diagnosis, investigations and treatment

No alternative neurological or orthopedic conditions explaining the upper limb spasticity were identified, and the findings were consistent with chronic post-stroke spastic paralysis.

A flossing intervention was applied to the affected right upper limb using an elastic floss band (COMPRE Floss^®^, 1-inch width, lime green; Sanct-Japan Inc.). The intervention was performed with the patient in a seated position. The flossing procedure, including circumferential wrapping of the distal forearm and active movements under compression, is illustrated in Figure 2. The floss band was wrapped circumferentially around the distal forearm extending to the elbow joint. Wrapping was initiated from the distal portion of the forearm and progressed proximally toward the elbow. The band was applied at approximately 1.5 times its natural length with an overlap of approximately 50% between each layer to provide uniform compression.

**Figure 2:**
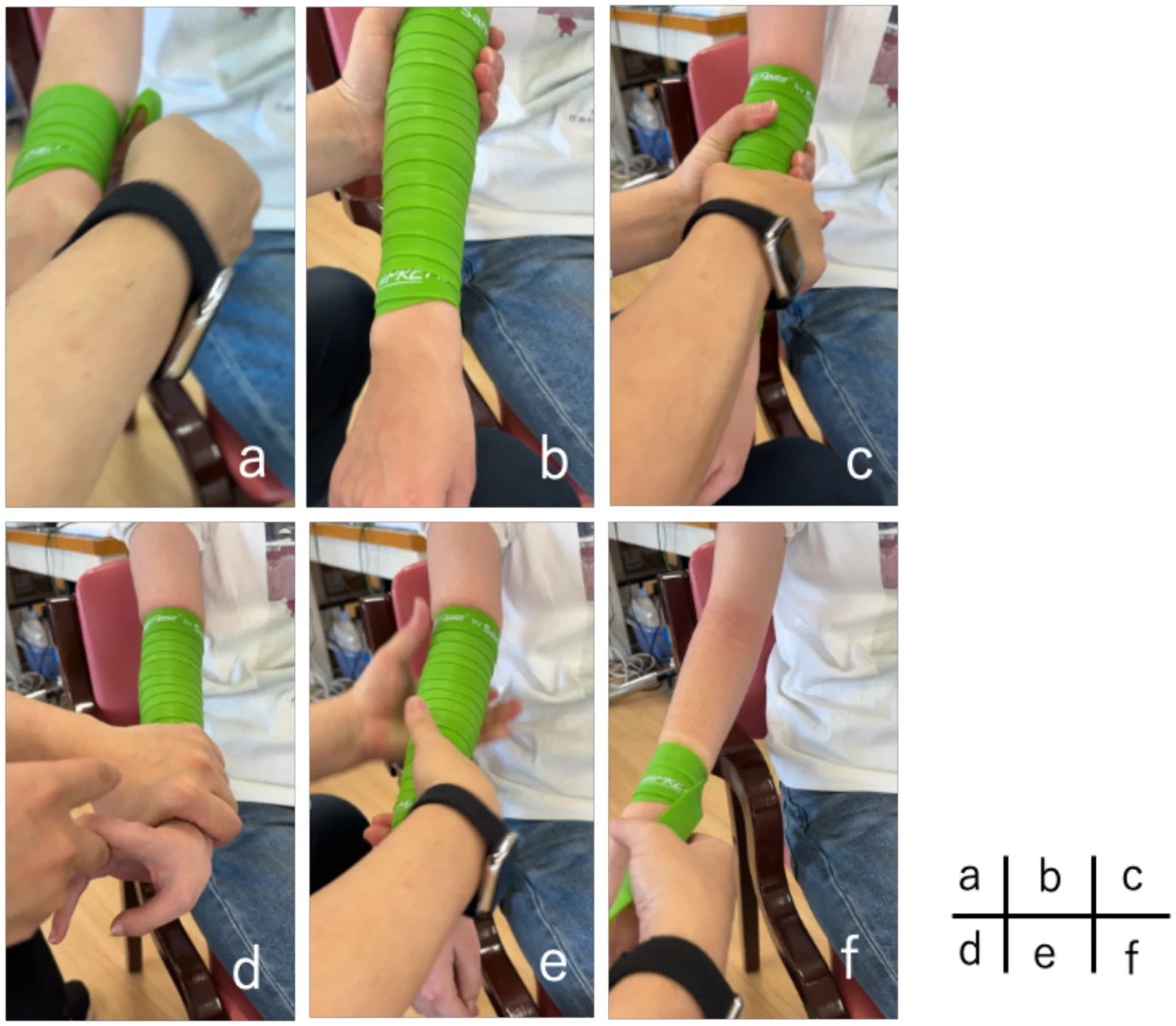
Floss band wrapping technique for the forearm and wrist. **(a)** The floss band was lightly wrapped once around the distal forearm to serve as an anchor and then applied proximally toward the elbow joint. **(b)** During wrapping, the band was stretched to approximately 1.5 times its resting length and overlapped by 50% with each turn. **(c)** The end of the band was tucked inward to secure fixation. **(d)** The wrapped area was compressed along its longitudinal axis for approximately 30 s and then twisted in a side-to-side direction for an additional 30 s. **(e)** While the band remained wrapped, a combination of active and passive movements was performed for approximately 1 min, including forearm pronation and supination, wrist flexion and extension, and finger flexion and extension. **(f)** After removal of the floss band, approximately 10 repetitions of active movements of the forearm, wrist, and fingers were performed without compression.

After wrapping, the wrapped region was manually compressed in the longitudinal direction for approximately 30 s and then twisted side-to-side for an additional 30 s. This was followed by a combination of active and passive movements under compression for approximately 1 min, including forearm pronation and supination, wrist flexion and extension, and finger flexion and extension. The floss band was then rapidly removed, followed by approximately 10 repetitions of active movements of the forearm, wrist, and fingers without compression. Including band application and removal, each set required approximately 4–5 min to complete. Two to three sets were performed per session, resulting in a total session duration of approximately 8–15 min.

At the initial outpatient occupational therapy session, the patient's mother was instructed in the floss band wrapping technique and exercise procedure for implementation as a home-based program. Because regular outpatient attendance was difficult owing to the patient's school schedule, supervised occupational therapy sessions were conducted approximately once every 2 months, coinciding with her neurosurgical follow-up visits, resulting in approximately three supervised sessions during the 6-month intervention period. According to reports from the patient's mother, the home-based flossing intervention was performed at least twice daily, typically in the morning and evening, with two to three sets performed during each session. Adherence to the home program was assessed through direct interviews with the patient's mother at each outpatient visit and by reviewing videos of the intervention performed at home. In addition, during outpatient sessions, the mother was asked to demonstrate the wrapping procedure so that the therapist could confirm the band tension and intervention technique. No formal exercise diary or other objective adherence-recording system was used. The intervention was continued for 6 months. During the 6-month intervention period, no other upper limb rehabilitation interventions, including electrical stimulation or additional exercise therapy, were provided. The patient did not receive botulinum toxin injections or other treatments for upper limb spasticity during this period. No changes in medication were made during the intervention period. No adverse events, such as increased pain, skin injury, or exacerbation of neurological symptoms, were observed during the intervention period.

## Conclusion and results (outcome and follow-up)

After 6 months of continuous flossing intervention, reductions in resistance to passive movement as assessed by the Modified Ashworth Scale, together with improvements in upper limb motor function and functional use of the affected hand, were observed. Quantitative changes in resistance to passive movement, upper limb motor function, range of motion, and real-world hand use before and after the intervention are summarized in Table 2. Modified Ashworth Scale scores decreased across all evaluated muscle groups. Improvements were also observed in upper limb motor function and active range of motion, with a particularly notable increase in wrist extension. Real-world use of the affected upper limb also increased, including both the amount and quality of use.

**Table 2:** Changes in spasticity, upper limb motor function, range of motion, and functional hand use before and after the flossing intervention.

Outcome measure	Baseline	After 6 months
Modified Ashworth scale		
Elbow flexors	1+	0
Forearm pronators	2	1+
Finger flexors	2	1
**Fugl–Meyer Assessment (upper limb)**	43 / 66	50 / 66
**Active wrist extension (degrees)**	−20°	40°
Motor activity log		
Amount of use (AOU)	1.42	2.21
Quality of movement (QOM)	1.71	2.64

MAS, Modified Ashworth scale; FMA, Fugl–Meyer Assessment; MAL, motor activity log. Wrist extension is expressed in degrees.

In practical activities, the patient was able to perform bilateral hand tasks that had been impossible prior to the intervention. In particular, fastening and unfastening buttons using both hands became possible for the first time since stroke onset. Improvements were also observed in movement speed and ease of manipulation during hand use.

The patient subjectively reported that her affected hand felt “easier to move,” and overall satisfaction with the intervention was high. Functional improvements were maintained throughout the follow-up period. Supplementary video materials demonstrate changes in wrist motion and bimanual task performance 6 months after the intervention (Supplementary Video 1). This case report describes functional improvement following a flossing intervention in an adolescent with long-standing upper limb spastic paralysis after childhood-onset stroke. Although limited to a single case, the intervention was associated with reductions in resistance to passive movement and improvements in motor function and real-world hand use. Given its non-invasive and home-based nature, flossing intervention may represent a feasible treatment option for selected patients with chronic post-stroke spastic paralysis. Further studies are warranted to confirm its efficacy and clarify appropriate indications.

## Discussion

In this case report, a flossing intervention was associated with reductions in resistance to passive movement as assessed by the Modified Ashworth Scale, together with improvements in upper limb motor function and functional hand use in an adolescent with long-standing upper limb spastic paralysis following childhood-onset stroke. Although causal relationships cannot be established from a single case, the observed changes were consistent across multiple outcome measures and were accompanied by meaningful improvements in activities of daily living.

One possible mechanism underlying the observed improvements is the combination of transient mechanical compression and subsequent changes in local tissue perfusion. Compressive wrapping followed by rapid removal of the floss band may alter local blood flow and tissue pressure. These changes may contribute to reduced resistance to passive movement and improved tissue mobility, thereby facilitating voluntary movement. However, the magnitude and clinical relevance of these circulatory responses have not been sufficiently established in neurological populations; therefore, improved circulation should be regarded as a possible contributing mechanism rather than a confirmed explanation for the observed reduction in resistance to passive movement. Another potential mechanism involves modulation of sensory and proprioceptive input (Zeng et al., 2023). Compression combined with active movement under pressure may stimulate cutaneous mechanoreceptors, muscle spindles, and Golgi tendon organs, thereby altering afferent input to the central nervous system (Proske and Gandevia, 2012). Similar neurophysiological mechanisms have been proposed in focal vibration therapy for post-stroke spasticity, in which repetitive mechanical stimulation is thought to modulate Ia afferent input, reduce spinal stretch-reflex excitability, and improve motor function (Caliandro et al., 2012; Noma et al., 2012; Giorgi et al., 2024; Nielsen et al., 2007). Recent systematic reviews have also suggested that peripheral mechanical stimulation may contribute to reductions in spasticity and improvements in upper limb motor performance after stroke, although the optimal stimulation parameters and underlying mechanisms remain uncertain (Zeng et al., 2023; Giorgi et al., 2024). Although flossing and focal vibration therapy both provide peripheral mechanical stimulation, they differ in stimulus characteristics and should not be assumed to act through the same afferent pathways. Therefore, the neurophysiological mechanisms proposed for focal vibration therapy cannot be directly extrapolated to flossing. Nevertheless, circumferential compression combined with movement during flossing may provide somatosensory input that could influence sensorimotor processing, although the specific neural mechanisms remain unclear. However, this hypothesis should be interpreted with caution because neurophysiological assessments, such as H-reflex or F-wave measurements, were not performed. Improved gliding of soft tissues, including muscles, tendons, and fascia, may also have contributed to functional gains. Previous studies in musculoskeletal populations have reported that flossing can improve range of motion and soft tissue mobility. In the present case, increased wrist extension range of motion and improved distal motor control may reflect enhanced mechanical properties of periarticular tissues. The reduction in Modified Ashworth Scale scores should be interpreted cautiously. The Modified Ashworth Scale assesses resistance to passive movement and does not distinguish between neural and non-neural components of increased muscle tone (Pandyan et al., 1999, 2003). Therefore, the observed reduction in scores may reflect not only changes in neural mechanisms but also changes in soft-tissue extensibility and mechanical resistance. Furthermore, because objective neurophysiological measures were not obtained, the observed changes in Modified Ashworth Scale scores cannot be considered definitive evidence of reduced neural spasticity.

To the best of our knowledge, this is among the first reports to describe functional improvement following a flossing intervention in a patient with post-stroke upper limb spastic paralysis (Konrad et al., 2021; Vogrin et al., 2021). Previous reports on flossing interventions have primarily focused on musculoskeletal conditions, and application to neurological disorders characterized by spasticity has rarely been described. Notably, the intervention was applied during the chronic phase following childhood-onset stroke, a period during which spontaneous neurological recovery is unlikely.

Furthermore, improvements were observed not only at the impairment level but also in real-world hand use, as assessed by the Motor Activity Log Uswatte et al. (2006) and supported by supplementary video documentation. These findings highlight the potential clinical relevance of flossing intervention beyond isolated biomechanical effects.

When applying flossing intervention, careful consideration of contraindications is essential because the technique involves external compression (Patterson et al., 2019). Absolute contraindications include active skin infection or dermatitis, varicose veins with inflammation, thrombosis, tumors, severe diabetes mellitus, fractures, open wounds, malignant neoplasms, heart failure, latex allergy, and the use of anticoagulant therapy or high-dose systemic corticosteroids.

Relative contraindications that require consultation with a physician include fever, pregnancy, hypertension, hypotension, chronic inflammatory conditions, significant psychological stress, and thyroid dysfunction.

In the present case, the patient was not receiving anticoagulant therapy or other medications associated with an increased risk of bleeding, and no contraindications were identified, allowing the safe implementation of the flossing intervention.

This case suggests that flossing intervention may be considered as an adjunctive option for managing chronic upper limb spasticity after stroke. The technique is simple, non-invasive, and feasible for home use when caregivers are appropriately instructed. Clinicians should carefully screen for contraindications and individual tolerance when applying compressive wrapping techniques.

Several limitations should be acknowledged. This report describes a single case without a control condition; therefore, a causal relationship between the flossing intervention and the observed improvements cannot be established. Although no other upper limb rehabilitation or spasticity-directed treatment was provided during the 6-month intervention period, the potential influence of unmeasured factors cannot be excluded. In addition, objective neurophysiological measures were not obtained, limiting mechanistic interpretation. Therefore, the findings should be interpreted cautiously. Future studies should aim to accumulate additional cases and evaluate the effects of flossing intervention using controlled study designs. Future studies incorporating neurophysiological measures (e.g., H-reflex, F-wave, or electromyography) may help clarify the specific neurophysiological effects of flossing and determine how they differ from those associated with other forms of peripheral mechanical stimulation, such as focal vibration. Investigations incorporating biomechanical and neurophysiological assessments may further clarify the mechanisms underlying changes in resistance to passive movement and motor function.

## Patient perspective

The patient reported being highly satisfied with the intervention. She stated that she was able to fasten buttons independently for the first time since the onset of stroke, which represented a meaningful improvement in her daily life. She also felt that bimanual activities had become much easier to perform. Both the patient and her mother expressed great satisfaction with the functional improvements achieved through the intervention. At the latest follow-up, she reported enjoying a fulfilling university life while actively using her affected upper limb in daily activities.

## Data Availability

The original contributions presented in the study are included in the article/Supplementary material, further inquiries can be directed to the corresponding author.
